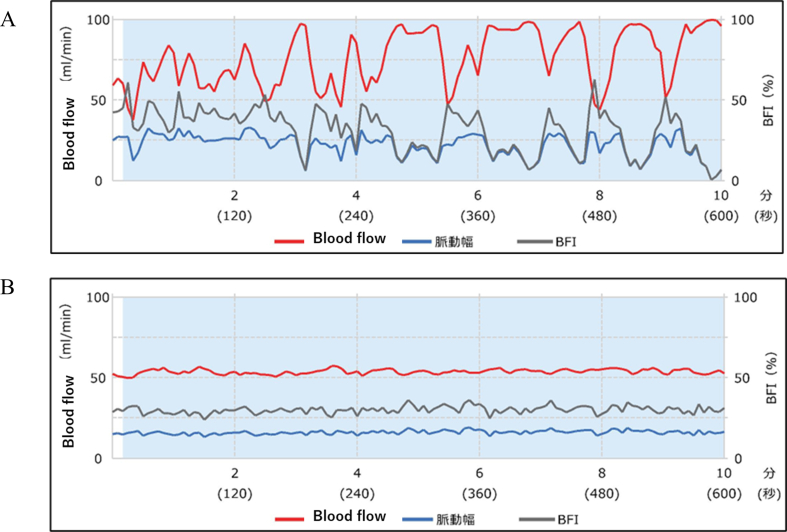# Feasibility of Portable Laser Doppler Flowmeter for Foot Blood Flow Assessment in Patients With Chronic Limb Threatening Ischaemia

**DOI:** 10.1016/j.ejvsvf.2025.09.003

**Published:** 2025-09-15

**Authors:** Masahiro Tezuka, Shotaro Hirota, Masashi Kawamura, Taisuke Konishi, Ikuko Shibasaki, Hirotsugu Fukuda

**Affiliations:** Department of Cardiac and Vascular Surgery, Dokkyo Medical University, Mibu, Tochigi, Japan

**Keywords:** Blood flow assessment, Chronic limb threatening ischaemia, Laser Doppler flowmetry, Lower extremity arterial disease, Revascularisation

## Abstract

**Objective:**

The portable, non-invasive laser Doppler flowmeter (LDF) provides a practical alternative, enabling bedside blood flow assessment for chronic limb threatening ischaemia (CLTI). The present study aimed to evaluate whether LDF could identify CLTI and to assess the utility of LDF in determining the efficacy of revascularisation.

**Methods:**

The study enrolled 50 patients with non–lower extremity arterial disease (LEAD) and 24 patients with CLTI (25 limbs) from March 2021 to December 2024; blood flow in the dorsal and plantar areas was measured using a portable LDF (Pocket LDF, JMS Co., Ltd., Tokyo, Japan). Blood flow and skin perfusion pressure (SPP) were compared before and after surgical bypass, and correlations between LDF and SPP were also determined for 17 limbs with CLTI.

**Results:**

The mean dorsal plantar blood flow was 12.0 ± 9.3 and 13.3 ± 6.6 mL/min for the non-LEAD and CLTI groups, respectively (*p* = .48), however, the mean plantar blood flow was statistically significant lower in the CLTI group (26.1 ± 11.7 mL/min) than in the non-LEAD group (52.2 ± 18.6 mL/min, *p* < .001). In the 17 limbs with CLTI that underwent surgical bypass, the mean blood flow in the dorsal area was 13.7 ± 5.5 and 15.7 ± 6.2 mL/min before and after surgery, respectively (*p* = .30). However, the post-operative mean plantar blood flow improved statistically significant (45.4 ± 18.6 mL/min) from its pre-operative status (25.0 ± 11.8 mL/min, *p* < .001). The mean SPP changes before and after surgical bypass were 27.3 ± 14.7 and 52.7 ± 18.3 mmHg, respectively (*p* < .001), in the dorsal area, showing improvement in correlation with the LDF measurements (*p* = .023).

**Conclusion:**

The portable LDF measurement in the plantar area may help in identifying CLTI and evaluating the effectiveness of revascularisation.

## Highlights

• Laser Doppler flowmetry was lower in chronic limb threatening ischaemia.
• The cut off value of chronic limb threatening ischaemia was 35.2 mL/min.
• After bypass surgery, laser Doppler flowmetry can assess improvement in blood flow.
• There is a correlation between laser Doppler flowmetry and skin perfusion pressure.

## INTRODUCTION

In various guidelines, the established blood flow assessment methods for lower extremity arterial disease (LEAD) include the ankle brachial index (ABI), toe brachial index, skin perfusion pressure (SPP), and transcutaneous oxygenation.^1^, ^2^, ^3^ However, these examinations are often challenging to perform because they require laboratory visits and can be time consuming. Patients with chronic limb threatening ischaemia (CLTI) may be difficult to examine because they may have ulcers or necrosis in their feet that prevent them from wearing the measuring device properly or they may have difficulty maintaining a resting position because of pain.

Laser doppler flowmetry, a real time, non-invasive method for measuring cutaneous microcirculation, has long been in clinical use^4^ and has been used to evaluate blood flow in patients with LEAD.^5^, ^6^, ^7^, ^8^ Recently, a portable laser doppler flowmeter (LDF) has been used clinically.^9^, ^10^, ^11^, ^12^, ^13^, ^14^ LDF enables the measurement of blood flow in capillaries at a depth of approximately 0.5 mm from the skin surface. The principle is that laser light irradiated onto the tissue shifts in frequency when it strikes an object (mainly red blood cells) moving within the capillaries (doppler effect); however, it does not change in frequency when it strikes stationary tissue. The velocity of the frequency shifted light returning to the receiver is proportional to the number of red blood cells, and the magnitude of the frequency shift is proportional to the blood flow velocity. Therefore, theoretically, blood flow calculation is possible by multiplying the red blood cell count and blood flow velocity.^15^^,^^16^

Although several studies have examined patients with LEAD using LDF,^5^, ^6^, ^7^, ^8^^,^^11^^,^^12^ few studies have been conducted on patients with CLTI or the diagnostic cutoff point. To date, no studies comparing blood flow before and after surgical bypass using LDF have been identified. This study aimed to evaluate whether a portable LDF could effectively identify CLTI by comparing the blood flow between patients without LEAD (non-LEAD) and those with CLTI. Moreover, the utility of LDF in determining the effectiveness of revascularisation was also examined by comparing the blood flow before and after bypass surgery in patients with CLTI.

## MATERIALS AND METHODS

### Study design

In this prospective observational study, the data of 50 patients with non-LEAD with 50 limbs and 24 patients with CLTI patients with 25 limbs who underwent surgery for various diseases at the Cardiac and Vascular Surgery Department of Dokkyo Medical University Hospital (Tochigi, Japan) from March 2021 to December 2024 were examined.

The non-LEAD group included patients admitted to the Cardiac and Vascular Surgery Department for operations other than LEAD. The inclusion criteria were as follows: normal ABI value (0.90–1.40), palpable dorsalis pedis or posterior tibial artery and without cardiac dysfunction (echocardiographic ejection fraction of >55%), considering the effect on lower extremity blood flow. For each patient, measurement of one limb was performed. The patient's sex and the location of the left or right lower extremity were not specified; however, to ensure that the patient's background matched the CLTI group, age was limited to >50 years. Additionally, imaging evaluation of lower extremity arteries was not included in the inclusion criteria. The CLTI group included patients with ulceration or gangrene in the lower extremity, ABI of <0.9, SPP value of <30, and ischaemia proven by contrast enhanced computed tomography or angiography and deemed to be requiring revascularisation to save the lower extremity. Furthermore, pre- and post-operative blood flow were compared in 17 CLTI limbs that underwent bypass surgery.

The present study was reviewed and approved by the Institutional Review Board of Dokkyo Medical University (11 October 2020; reference number: R-39-8J). Additionally, informed consent was obtained from all study participants.

### LDF measurement

Foot blood flow was measured using a commercially available portable LDF (Pocket LDF, JMS Co., Ltd., Tokyo, Japan). The Pocket LDF is a compact device (105 × 62 × 25 mm, 144 g) that allows for easy portability. The sensor probe (12 × 3 mm, 21 g) is composed of the laser emitting and backscattered light receiving units and is provided with a window on the surface contacting the body. The main unit is equipped with a battery and wireless transmission function, and results can be recorded on a personal computer.^9^^,^^16^

The non-LEAD group underwent one measurement during hospitalisation. The CLTI group underwent measurements before revascularisation in the absence of ulceration or gangrene on the affected limb. In the surgical group, post-operative LDF measurement was performed approximately two weeks after surgery, with the exclusion of cases in which the graft was occluded before measurement or in cases where the graft was amputated owing to uncontrollable infection.

The measurement sites were the dorsal and plantar portions of the foot, as in SPP, which is also a laser doppler based test. The specific measurement sites were the base of the first toe and ball of the foot. The blood flow of the dorsal and plantar areas was measured simultaneously in all patients using two portable LDF devices (Supplementary Figure S1). During the measurement, the patient rested in a supine position and maintained the same posture for 10 minutes. All cases were measured by the single examiner (M.T.).

Skin blood flow is affected by external factors, including room temperature, humidity, airflow, and season, as well as internal factors, such as limb position, sweating, and fever;^17^ therefore, the setting of environmental conditions should be considered. In the present study, to eliminate measurement bias as much as possible, the measurements were performed on hospitalised patients to remove the external factors and the patients were placed in the supine position to eliminate the internal factors.

### Statistical analysis

The Shapiro–Wilk test was used to assess the normality of continuous variables. Continuous variables were presented as mean ± standard deviation or median (interquartile range), and categorical variables were presented as counts and percentages. For normally distributed continuous variables, a two tailed Student's *t* test or Fisher's exact test was used. For the continuous variables that were not normally distributed, the Mann–Whitney *U* test was used. Categorical variables were assessed using the chi squared test or Fisher's exact test. Pearson's correlation coefficient was used to assess the linear relationship between the LDF and SPP values, assuming normally distributed data. Receiver operating characteristic (ROC) curve analysis was performed to determine the optimal cutoff point for diagnosing patients with CLTI using portable LDF. The optimal cutoff value was determined using Youden's index, which maximises the sum of sensitivity and specificity. The sensitivity, specificity, and false positive rate (1- specificity) were calculated to generate the ROC curve. All *p* values were two sided and *p* values of ≤.050 were considered statistically significant. Statistical analyses were performed with SPSS Statistics 30.0 (IBM, Armonk, NY, USA) and EZR (Saitama Medical Centre, Jichi Medical University, Saitama, Japan), which is a graphical user interface for R (The R Foundation for Statistical Computing, Vienna, Austria).^18^

## RESULTS

### Patient characteristics

The patients’ characteristics of the non-LEAD and CLTI groups were compared (Table 1). The prevalence of diabetes (26.0% *vs.* 79.2%, *p* < .001), dialysis (0% *vs*. 41.7%, *p* < .001) and coronary artery disease (4.0% *vs*. 41.7%, *p* < .001) was statistically significant higher in the CLTI group than in the non-LEAD group.

**Table 1:** Clinical characteristics of patients with non–lower extremity arterial disease (LEAD) and patients with chronic limb threatening ischaemia (CLTI).

Variable	Label	Non-LEAD (*n* = 50)	CLTI (*n* = 24)	*p* value
Age – y		73.5 (68.0–77.0)	75.0 (67.3–78.3)	.72
Male, *n* (%)		36 (72.0)	19 (79.2)	.58
ABI∗		1.1 (1.1–1.2)	0.6 (0.2–0.8)	<.001
Reason for surgery, *n* (%)	Aneurysm	42 (84.0)	0 (0)	<.001
	Aortic dissection	1 (2.0)	0 (0)	1.0
	Valve disease	4 (8.0)	0 (0)	.30
	Angina pectoris	1 (2.0)	0 (0)	1.0
	Other cardiac disease	2 (4.0)	0 (0)	1.0
	CLTI	0 (0)	24 (100)	<.001
Measured limbs∗, *n* (%)	Right	27 (54.0)	13 (52.0)	1.0
	Left	23 (46.0)	12 (48.0)	
Ejection fraction∗ – %		62.0 (60.0–65.0)	60.0 (47.0–64.0)	.004
Hypertension, *n* (%)		37 (74.0)	16 (66.7)	.59
Dyslipidaemia, *n* (%)		21 (42.0)	8 (33.3)	.61
Diabetes, *n* (%)		13 (26.0)	19 (79.2)	<.001
Dialysis, *n* (%)		0 (0)	10 (41.7)	<.001
COPD, *n* (%)		4 (8.0)	0 (0)	.30
CAD, *n* (%)		2 (4.0)	10 (41.7)	<.001
CVD, *n* (%)		6 (12.0)	5 (20.8)	.32

Data are presented as median (interquartile range) unless stated otherwise. ABI = ankle–brachial index; CAD = coronary artery disease; CLTI = chronic limb threatening ischaemia; COPD = chronic obstructive pulmonary disease; CVD = cerebrovascular disease; LEAD = lower extremity arterial disease.

∗ ABI, measured limbs, and ejection fraction of patients with CLTI were calculated on the basis of 25 limbs.

The details of the CLTI group are described in Table 2. All patients with CLTI were classified as Fontaine stage IV, with 68.0% of the patients having gangrene and 32.0% having ulcers. Most patients had diseased arteries in the lower leg, most commonly in the peroneal (76.0%) and anterior tibial (72.0%) arteries. The majority of treatments were surgical bypasses, which was performed in 72.0% of cases.

**Table 2:** Data of patients with chronic limb threatening ischaemia (CLTI).

Variable	Label	CLTI limbs (*n* = 25)
SPP – mmHg	Dorsal	27.6 ± 13.9
	Plantar	24.7 ± 13.6
Fontaine classification stage	Ⅲ	0 (0)
	*Ⅳ*	
	Ulcer	8 (32.0)
	Gangrene	17 (68.0)
Diseased limb	Right	13 (52.0)
	Left	12 (48.0)
Diseased vessel	Aorta	0 (0)
	Iliac	3 (12.0)
	Femoral	12 (48.0)
	Popliteal	10 (40.0)
	Anterior tibial	18 (72.0)
	Peroneal	19 (76.0)
	Posterior tibial	12 (48.0)
Revascularisation method	Endovascular treatment	1 (4.0)
	Surgical bypass	22 (72.0)
	Endarterectomy	1 (4.0)
	Conservative treatment	1 (4.0)

Data are presented as mean ± standard deviation or *n* (%). CLTI = chronic limb threatening ischaemia; SPP = skin perfusion pressure.

### Comparison of the non-LEAD and CLTI groups

In the comparison between 50 non-LEAD and 25 CLTI limbs, the mean dorsal blood flow was 12.0 ± 9.3 and 13.3 ± 6.6 mL/min, respectively (*p* = .48). However, the mean plantar blood flow was statistically significant lower in the CLTI group (26.1 ± 11.7 mL/min) than in the non-LEAD group (52.2 ± 18.6 mL/min, *p* < .001) (Fig. 1A).

**Figure 1:**
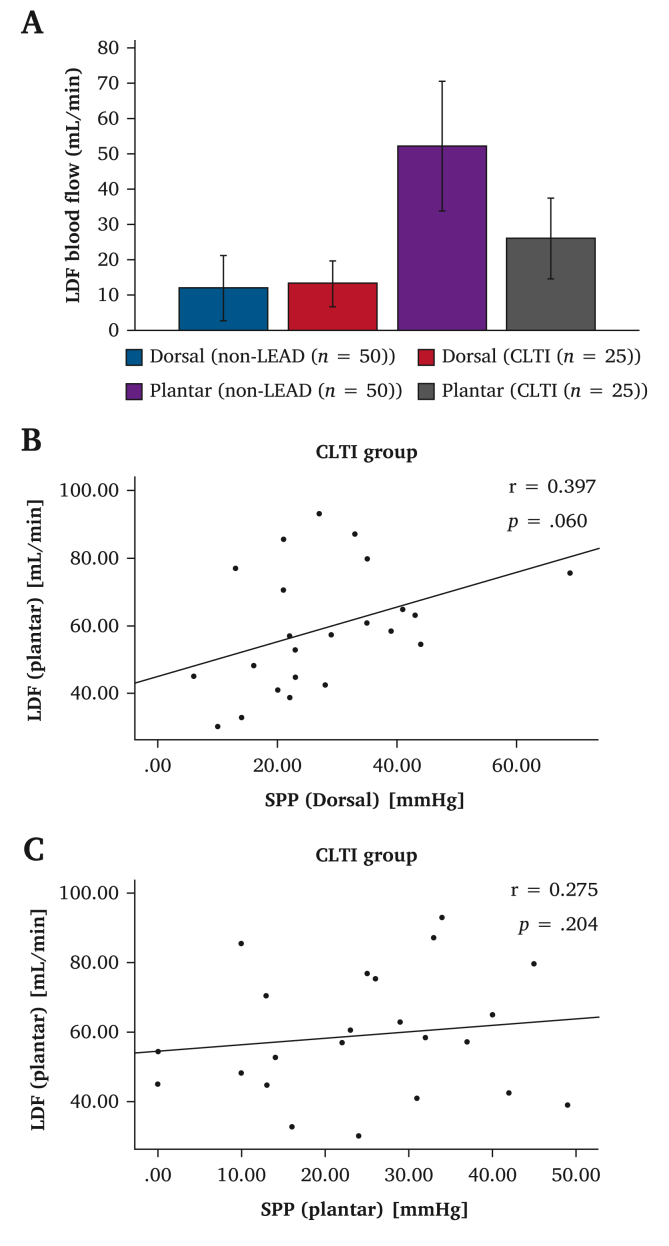
Comparison of the non–lower extremity arterial disease (LEAD) and chronic limb threatening ischaemia (CLTI) groups. A Comparison of portable laser Doppler flowmeter (LDF) blood flow values in the non-LEAD and CLTI groups. B Scatter plots and correlation coefficients between plantar LDF blood flow and dorsal skin perfusion pressure (SPP) values. C Scatter plots and correlation coefficients between plantar LDF blood flow and plantar SPP values.

In the CLTI group, the mean SPP values were 27.6 ± 13.9 and 24.7 ± 13.6 mmHg in the dorsal and plantar areas, respectively. The correlation coefficient between the plantar LDF and dorsal SPP values was 0.397 (*p* = .060) (Fig. 1B), and that between the plantar LDF and plantar SPP values was 0.275 (*p* = .20) (Fig. 1C). The correlation coefficient between the dorsal and plantar SPP values was also 0.275 (*p* = .20).

An ROC curve analysis of the dorsal and plantar area measurements of the non-LEAD and CLTI groups was performed.

In the dorsal area, the LDF blood flow of 9.3 mL/min was the cutoff point for CLTI (area under the curve [AUC] 0.62), with a sensitivity of 49.6% and specificity of 75.1% (Fig. 2A). In the plantar area, the LDF blood flow of 35.2 mL/min was the cutoff point for CLTI (AUC 0.89), both with a sensitivity and specificity of 84.0% (Fig. 2B).

**Figure 2:**
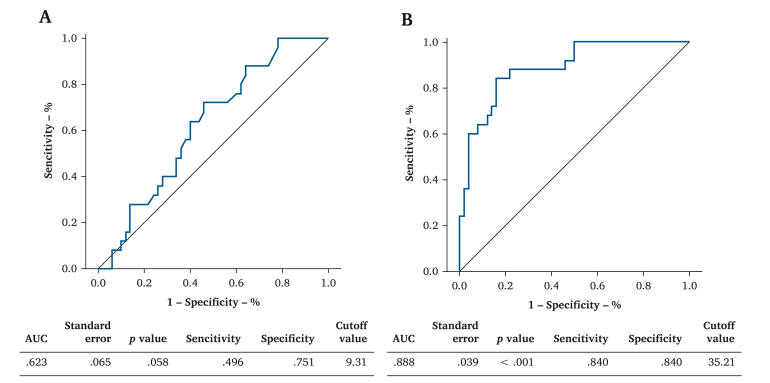
Receiver operating characteristic (ROC) analysis. A ROC curve, cutoff point, and area under the curve (AUC) for the dorsal area. B ROC curve, cutoff point, and AUC for the plantar area.

The mean blood flow at one, three, five, and 10 minutes in the non-LEAD group was 11.7 ± 8.5, 11.7 ± 8.8, 11.7 ± 8.9, and 12.0 ± 9.3 mL/min, respectively, on the dorsal area and 49.0 ± 17.9, 51.6 ± 18.1, 51.1 ± 17.9, and 52.2 ± 18.6 mL/min, respectively, on the plantar area. In the dorsal region, no statistically significant differences were observed at each time point (*p* > .050). In the plantar area, statistically significant differences were noted at one and three minutes (*p* = .005), but no statistically significant differences were found after three minutes (*p* > .050) (Supplementary Figure S2).

### Comparisons in the surgical group

**Figure 3:**
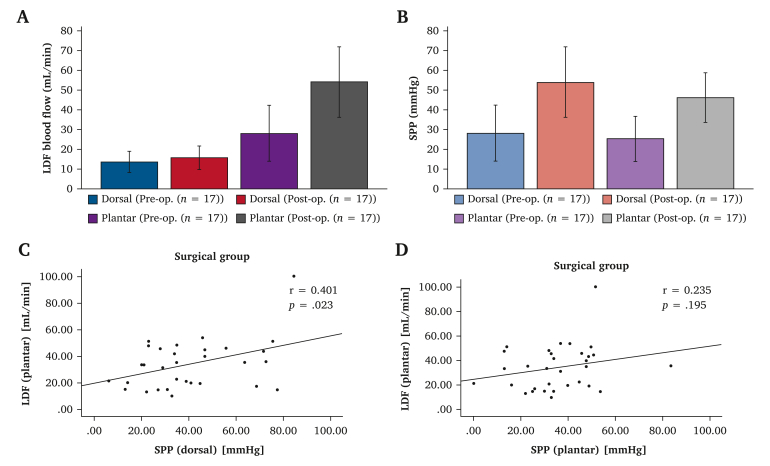
Comparison of the surgical group. A Comparison of pre-operative (Pre-Op.) and post-operative (Post-Op.) portable laser Doppler flowmeter (LDF) blood flow values. B Comparison of Pre-Op. and Post-Op. skin perfusion pressure (SPP) values. C Scatter plots and correlation coefficients between plantar LDF blood flow and dorsal SPP values. D Scatter plots and correlation coefficients between plantar LDF blood flow and plantar SPP values.

Pre- and post-operative blood flows were compared for the 17 CLTI limbs that underwent bypass surgery. The mean pre- and post-operative dorsal blood flow was 13.7 ± 5.5 and 15.7 ± 6.2 mL/min, respectively (*p* = .30). However, the mean plantar blood flow was statistically significant improved after surgery (45.4 ± 18.6 mL/min) compared with the value before surgery (25.0 ± 11.8 mL/min, *p* < .001) (Fig. 3A).

The SPP values were obtained both before and after surgery, yielding an average of 27.3 ± 14.7 and 52.7 ± 18.3 mmHg, respectively (*p* < .001), for the dorsal area and 24.3 ± 11.6 and 43.6 ± 8.2 mmHg, respectively (*p* < .001), for the plantar area (Fig. 3B).

The pre- and post-operative correlation coefficient between the plantar LDF and dorsal SPP values was 0.401 (*p* = .023) (Fig. 3C) and that between plantar LDF and plantar SPP values was 0.235 (*p* = .20) (Fig. 3D). The correlation coefficient between the dorsal and plantar SPP values was 0.776 (*p* < .001).

## DISCUSSION

To summarise the study results, the portable LDF blood flow measurements in the dorsal area did not differ between patients with non-LEAD and patients with CLTI (*p* = .48). Conversely, in the plantar area, the patients in the non-LEAD group had greater blood flow than the patients with CLTI (*p* < .001). The ROC curve analysis revealed that a cutoff value of 35.2 mL/min in the plantar area (AUC 0.89) could help to differentiate patients with CLTI from those with non-LEAD, with the plantar area showing a higher diagnostic accuracy. The improvement in blood flow was observed only in the plantar area before and after surgical bypass for patients with CLTI (*p* < .001). A statistically significant improvement was observed in both the dorsal and plantar SPP values before and after surgery. Moreover, a statistically significant correlation was identified between the plantar LDF and dorsal SPP values (*p* = .023).

In the dorsal and plantar LDF measurements, the only discrepancy in blood flow observed between the non-LEAD and CLTI groups was in the plantar area. Moreover, even after the surgical bypass for CLTI, the improvement in blood flow was only observed in the plantar area. Like this study, several studies have compared dorsal and plantar blood flow with LDF measurements, and all studies have reported that the plantar blood flow is higher than the dorsal blood flow. This difference may be due to various reasons.^12^^,^^19^^,^^20^ In a previous study involving rats, Rendell *et al.*^20^ found higher vascular density and vascular volume in the plantar area than in the dorsal area. Ishii *et al.*^12^ compared patients with non-LEAD and patients with LEAD on haemodialysis with portable LDF, and consistent with the results of this study, showed that the dorsal blood flow was not statistically significant different between the two groups; however, the plantar blood flow was statistically significant lower in the LEAD group. It was considered that the difference was due to the fact that the dorsal side has more tendon and hair root follicle and lesser micro blood flow as compared with the plantar side.^12^ These results suggest that the dorsal area may have a low vascular density, making the measurement of blood flow with a small LDF probe difficult. This may explain why the blood flow was lower in the non-LEAD and CLTI groups and in patients who underwent surgical bypass for CLTI.

Another reason may be the measurement depth of the portable LDF. The thickness of the epidermis is approximately 0.2 and 0.6 mm on the dorsal and plantar sides, respectively.^21^ Beneath the epidermis is the dermis, which contains capillaries in the uppermost layer and arterioles in the lower layer.^17^^,^^22^^,^^23^ Given that the portable LDF measures blood flow at a depth of approximately 0.5 mm from the surface, the blood flow on the dorsal area reflects capillaries in the uppermost dermal layer and arterioles in the lower layer. In contrast, blood flow may only be reflected in the capillaries in the plantar area. Although the portable LDF is designed to measure the flow velocities in the low velocity range of 1–10 mm/sec,^16^ arteriolar flow velocities are faster than 10 mm/sec^24^ and may not be measured well by the portable LDF. This suggests that the LDF is measuring the depth of the arteriole in the dorsal area, which would lead to inaccurate results and may be the reason for the absence of a difference in the measurements between the non-LEAD and CLTI groups. In the plantar area, however, portable LDF was suggested not only to allow differentiation between patients with non-LEAD and patients with CLTI but also to be a sensitive indicator of improved blood flow after revascularisation.

In the present study, SPP was measured for the CLTI and surgical groups. The mean pre-operative SPP value of the CLTI group was <30 mmHg, which is below the limit for anticipated wound healing. After surgery, a marked improvement was observed in the dorsal and plantar areas. The only statistically significant correlation was observed between the plantar LDF and dorsal SPP values. The absence of correlation between the plantar LDF and plantar SPP values may be attributed to the sample size, given that a correlation between the dorsal and plantar SPP values was observed in the surgical group. The measurement of SPP, like LDF, is a laser doppler based assessment method. A direct comparison between these two examinations is precluded because SPP measures blood pressure rather than blood flow. However, the correlation between SPP, an established test for patients with CLTI, and LDF results suggests that the blood flow measurements obtained with the portable LDF are reliable.

Although it may be possible to differentiate patients with non-LEAD from patients with CLTI by determining the blood flow in the plantar area, the specific blood flow for diagnosing of CLTI remains unknown. To determine the cutoff point for CLTI, an ROC curve was obtained for the non-LEAD and CLTI groups and a plantar blood flow of 35.2 mL/min was the cutoff point for CLTI (AUC 0.89), both with a sensitivity and specificity of 84.0%.

In previous studies, the appropriate measurement time for LDF has not been described. The actual measurement results show that the changes in blood flow over time vary from person to person, with some people experiencing large fluctuations (Supplementary Figure S3A) and others experiencing slight fluctuations (Supplementary Figure S3B). To determine the appropriate measurement time for the negligible measurement error, the mean blood flow at the first one, three, five, and 10 minutes of a 10 minute measurement in the non-LEAD group was compared. A statistically significant difference was observed between the one and three minute measurements in the plantar area (*p* = .005), but no statistically significant difference was observed after these time points. These results suggest that measurements of at least three minutes are sufficiently reliable, even if there are fluctuations in blood flow over time in the individual patients. Blood flow stabilised after three minutes, suggesting that shorter measurement durations are feasible and clinically practical, particularly in situations where rapid and straightforward assessment is required, such as at the bedside or in the outpatient setting.

The present study has several limitations. Firstly, it was conducted at a single centre, possibly introducing patient selection bias. Secondly, some patients with non-LEAD included in this study may still have underlying atherosclerotic disease, potentially affecting the measurement accuracy. Thirdly, the sample size was relatively small, which may limit the findings’ statistical power. Fourthly, the variations in measurement environments, such as the outpatient or emergency settings, may affect the reproducibility of the results. In the future, conducting a multicentre study to secure a more significant number of cases and eliminating patient bias would be desirable. Although this study demonstrated that surgical bypass in patients with CLTI resulted in improved blood flow in the plantar area, it did not examine whether this improvement led to wound healing. Moreover, numerous factors other than blood flow contribute to wound healing in CLTI, and enhanced blood flow does not necessarily lead directly to wound healing.^25^, ^26^, ^27^, ^28^, ^29^ As with SPP and transcutaneous oxygenation,^30^, ^31^, ^32^, ^33^ further studies are needed to determine the cutoff value at which wound healing is achieved.

## CONCLUSION

The present study has shown that portable LDF measurement in the plantar area may help identify patients with CLTI requiring revascularisation. A cutoff value of 35.2 mL/min for plantar blood flow showed high diagnostic accuracy, highlighting potential clinical utility. It is also helpful in determining the effectiveness of revascularisation because of its sensitivity to improved blood flow before and after surgical bypass.

## Funding

This research did not receive any specific grant from funding agencies in the public, commercial, or not for profit sectors.

## DATA STATEMENT

Data are not available owing to privacy restrictions.

## CONFLICT OF INTEREST

None.

## Figures and Tables

**figs1:**
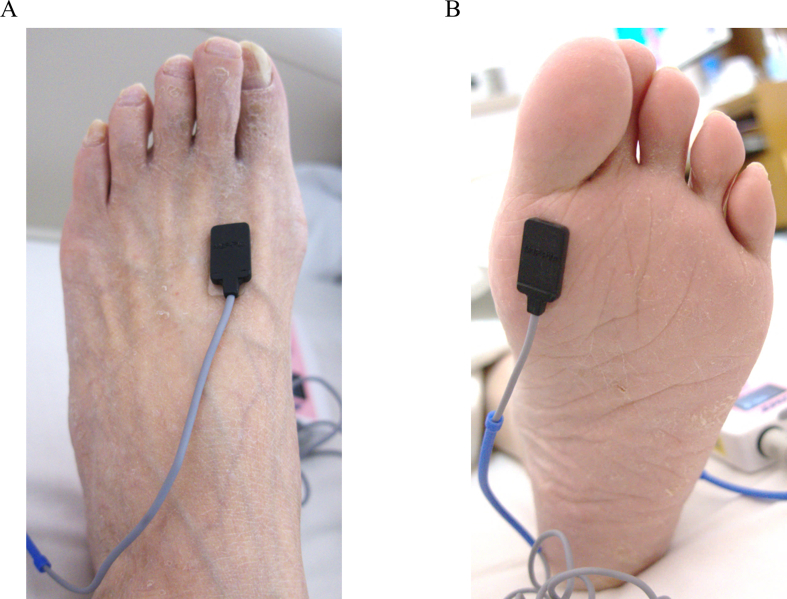


**figs2:**
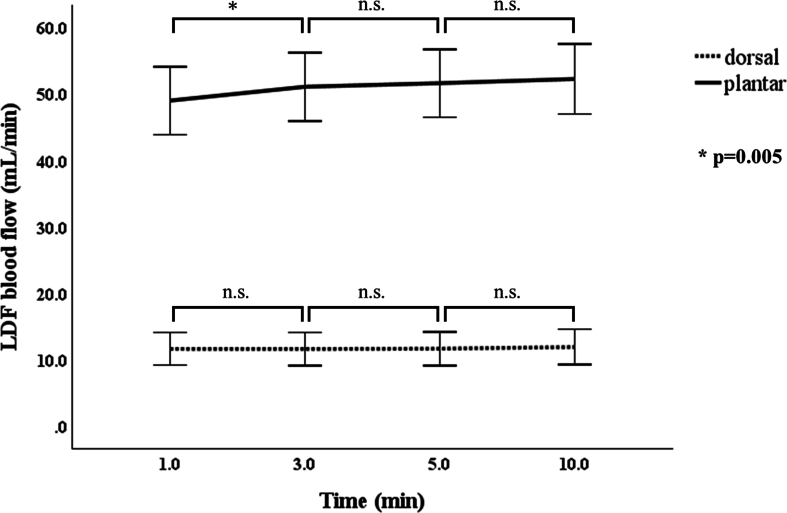


**figs3:**