# Supplementary material for: Feasibility of Portable Laser Doppler Flowmeter for Foot Blood Flow Assessment in Patients With Chronic Limb Threatening Ischaemia
Source: EJVES Vasc Forum. 2025 Sep 15;64:166–73. doi: 10.1016/j.ejvsvf.2025.09.003 (PMC12557605; doi:10.1016/j.ejvsvf.2025.09.003)
Supplement: Multimedia component 1 [file mmc1.docx]

**Supplementary Figure legends**

Supplementary Figure S1: Mounting site for the portable laser Doppler flowmeter (LDF)

A. Dorsal area (base of the first toe)

B. Plantar area (ball of the toe)

Supplementary Figure S2: Mean blood flow by measurement time

Each plot is indicated as mean blood flow + 95% confident interval

Supplementary Figure S3: Individual variation in blood flow changes over time in portable laser Doppler flowmeter (LDF)

A: Patients with marked fluctuations in blood flow over time

B: Patients with slight fluctuations in blood flow over time
